# High prevalence of mutations affecting the splicing process in a Spanish cohort with autosomal dominant retinitis pigmentosa

**DOI:** 10.1038/srep39652

**Published:** 2017-01-03

**Authors:** Maitane Ezquerra-Inchausti, Olatz Barandika, Ander Anasagasti, Cristina Irigoyen, Adolfo López de Munain, Javier Ruiz-Ederra

**Affiliations:** 1 Division of Neurosciences, Biodonostia Health Research Institute, San Sebastián, Spain; 2 Department of Ophthalmology, Donostia University Hospital, San Sebastián, Spain; 3 Department of Neurology, Donostia University Hospital, San Sebastián, Spain; 4 CIBERNED, Center for Networked Biomedical Research on Neurodegenerative Diseases, Institute of Health Carlos III, Ministry of Economy and Competitiveness, Spain; 5 Department of Neurosciences, University of the Basque Country UPV-EHU, Spain

## Abstract

Retinitis pigmentosa is the most frequent group of inherited retinal dystrophies. It is highly heterogeneous, with more than 80 disease-causing genes 27 of which are known to cause autosomal dominant RP (adRP), having been identified. In this study a total of 29 index cases were ascertained based on a family tree compatible with adRP. A custom panel of 31 adRP genes was analysed by targeted next-generation sequencing using the Ion PGM platform in combination with Sanger sequencing. This allowed us to detect putative disease-causing mutations in 14 out of the 29 (48.28%) families analysed. Remarkably, around 38% of all adRP cases analysed showed mutations affecting the splicing process, mainly due to mutations in genes coding for spliceosome factors (SNRNP200 and PRPF8) but also due to splice-site mutations in RHO. Twelve of the 14 mutations found had been reported previously and two were novel mutations found in PRPF8 in two unrelated patients. In conclusion, our results will lead to more accurate genetic counselling and will contribute to a better characterisation of the disease. In addition, they may have a therapeutic impact in the future given the large number of studies currently underway based on targeted RNA splicing for therapeutic purposes.

Retinitis pigmentosa (RP; MIM# 268000) is the most frequent form of inherited retinal dystrophy (IRD), with a prevalence of 1 in 3000–4000 cases worldwide^1^. It is characterised by a progressive dysfunction associated with the death of rods and/or cones, which leads to retinal atrophy and loss of vision. The mode of inheritance of RP is complex, with autosomal dominant (ad), autosomal recessive (ar), X-linked (xl) Mendelian cases and some cases of digenism or mitochondrial forms having been reported^1^,^2^,^3^. From a genetic perspective, over 80 disease-causing genes are currently associated with RP, 27 of which have been associated with adRP (http://www.sph.uth.tmc.edu/retnet). However, to date, mutations in the known adRP genes account for only 50–75% of dominant cases, depending on the test and population used in the study^4^. This percentage is increasing, mainly due to the implementation of Next Generation Sequencing (NGS)-based techniques^5^,^6^,^7^ and the discovery of new RP genes^8^,^9^,^10^,^11^.

Most human genes harbour introns that are removed during pre-mRNA splicing post-transcriptional modification^12^. The splicing reaction is catalysed by the spliceosome, a multisubunit complex comprising small noncoding nuclear RNAs (U1, U2, U4, U5, and U6) and several associated proteins^13^. The spliceosome orchestrates the two transesterification reactions needed to remove introns and to join the adjacent exons, and operates by step-wise formation of sub-complexes that recognise regulatory sequences and promote efficient splicing^12^,^13^,^14^.

Mis-regulation of splicing is a common feature of many human diseases, including several retinal diseases^15^,^16^,^17^,^18^. These disorders can be caused by mutations that disrupt the splicing of specific genes or by mutations in genes coding for splicing factors, both of which lead to a general loss of spliceosomal function. Thousands of splice-site mutations have been identified in patients with retinal dystrophies. Although most of these mutations disrupt a consensus splice-site sequence and cause exon skipping, some result in intron inclusion, novel exon inclusion, or the usage of cryptic upstream or downstream splice sites. The resulting alteration in the protein sequence, which is often concomitant with frameshift and premature termination, unsettles the functional protein domains and leads to degeneration of the retina^16^. For example, mutations in several genes coding for core spliceosomal proteins, such as pre-mRNA splicing factors (PRPF3, PRPF4, PRPF6, PRPF8, PRPF31, RP9) or RNA helicases (SNRNP200), are responsible for adRP^14^,^16^,^17^. However, given that these genes are expressed ubiquitously in all tissues and are highly conserved in all eukaryotes, it remains unclear why mutations in these genes are associated exclusively with adRP. Studies performed in rodent retina showed that PRPF3, PRPF31, PRPC8 expression levels are higher in the retina than in other tissues in normal adult mice, thus suggesting that the retina may have a higher basal splicing demand than other tissues given that it is one of the most metabolically active tissues in the body^16^,^19^.

In order to effectively identify adRP mutations, we have sequenced 31 genes associated with the autosomal dominant inheritance pattern using the Ion PGM platform (IPGM; Life Technologies), in combination with Sanger sequencing. We selected these genes as they have been linked to most of the cases of adRP reported. Remarkably, we found a high prevalence of mutations affecting the splicing process among our families, especially mutations affecting *trans*-acting splicing factors. This is of particular interest considering that several splicing-based therapeutic approaches, some of which are in clinical trials^15^,^17^, are under active development for mutations affecting either core spliceosomal proteins or splice site mutations of individual genes.

The results of the present study will help in genetic counselling and will contribute to a better characterisation of the disease. Moreover, they may have a therapeutic impact in the near future in the light of analogous approaches used for other RNA mis-splicing diseases.

## Results

### High variant detection coverage and sensitivity was achieved

An average of 3.3 million reads/chip was obtained. On average, each amplicon present in the panel was covered 658 times, with 95.92% of amplicons with >30x coverage and 94.27% of amplicons with >50x coverage. Those regions with no or low coverage (<30X), probably due to the presence of repetitive sequences or self-annealing of primers, were re-analysed. A highly sensitive, cost-effective method described recently by us that combines high resolution melting (HRM) analysis with direct sequencing was used for this re-analysis^20^. This allowed us to expand our analysis to 97% of target amplicons. Despite the implementation of HRM, no additional mutations were found within these re-analysed regions.

### Variant identification

An average of 45 variants, including SNPs and INDELS, were initially identified for each sample in the targeted regions, including the negative control with 51 SNPs, none of which were putative disease-causing as expected (see Supplementary Table S1). After the clinically relevant variant identification screening described in the materials and methods section, we were able to identify putative disease-causing mutations in a total of 14 out of the 29 probands, which resulted in a ratio of clinically relevant genetic findings of 48.28%. A description of the main features of the genetic findings can be found in Table 1.

**Figure 1:**
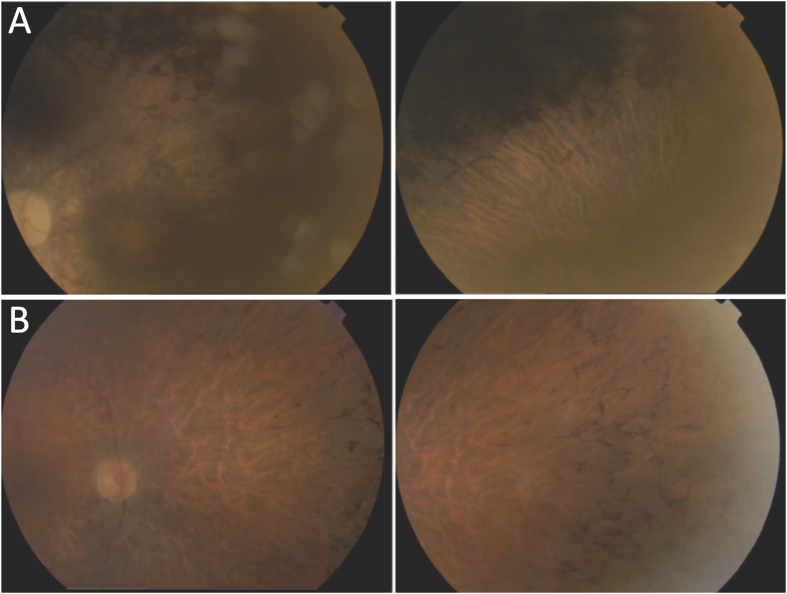
Fundus photographs of patients with novel mutations in PRPF8. (**A**) Patient RP90 (p.Val2325_Glu2330del) shows optical disc pallor, arteriolar attenuation and macular atrophy (right), with dense pigment in the mid-periphery (left). (**B**) Patient RP148 (p.Leu2315Leufs*2336Aspext*21) shows optical disc pallor, arteriolar attenuation and bone spicule-shaped pigment deposits in the mid-periphery. The left and right pictures correspond to the left and right eyes, respectively.

A total of seven variants in four genes were found in 14 families. Two of these mutations (both in PRPF8) were novel and were found in two families. One consisted in a loss of 21 nucleotides (p.Val2325_Glu2331del) and the other consisted of a frameshift deletion involving a single-point deletion (p.Leu2315Leufs*2336Aspext*21). Figure 1 shows colour fundus pictures of patients RP90 and RP113 bearing these two novel mutations. Both novel variants were potentially pathogenic, co-segregated with the disease, and were predicted as pathogenic by MutationTaster.

Two genes were involved in 37.93% of our cohort of families, with RHO affecting four probands with three different mutations and SNRNP200 affecting seven probands, all with the p.Ser1087Leu mutation^21^,^22^.

The high prevalence of mutations affecting the splicing process among our families (11 out of 29 probands studied), representing 38% of the probands in our adRP cohort, was unexpected. Most cases (9/29) were due to mutations affecting the genes SNRNP200 (7) and PRPF8 (2), which code for core spliceosomal proteins, although a splice site mutation in RHO^23^ was also detected (2/29).

**Figure 2:**
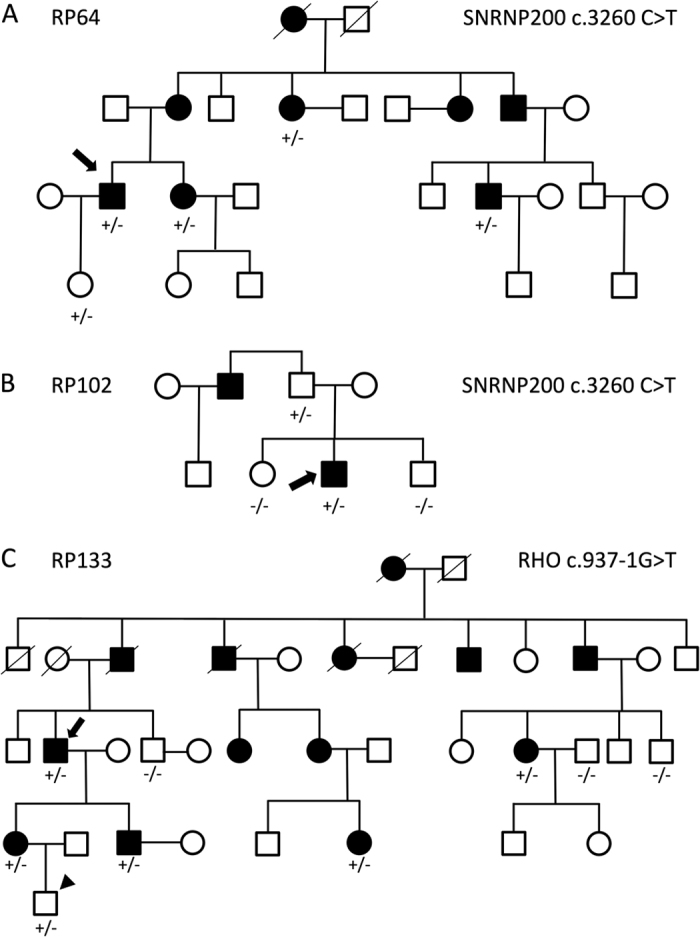
Representative trees for families with the two most prevalent mutations found in SNRNP200 and RHO genes. The p.Ser1087Leu mutation in SNRNP200 was found in families RP64 (**A**) and RP102 (**B**). (**C**) The c.937-1G > T mutation in the RHO splice acceptor site in a total of six individuals from family RP133, one of whom is a young asymptomatic patient (arrowhead). Genotypes are annotated as +/− (heterozygote) or −/− (wild type). Arrows indicate proband patients.

With respect to SNRNP200, after performing Sanger sequencing in all available family members we identified c.3260C > T mutation in a total of 12 cases from seven families (see representative family in Fig. 2A). Co-segregation analysis showed that two out of seven healthy subjects analysed for this variant in these families were mutation carriers, which likely indicates cases of incomplete penetrance similar to what has recently been reported for this variant in a study also involving a Spanish cohort^7^ (see Fig. 2B). We also found a total of nine individuals in two families with c.937-1G > T mutations affecting RHO splicing. Interestingly, one of these nine patients is asymptomatic, probably due to the disease being in an initial state given his young age (21 years old; see Fig. 2C and Supplementary Fig. S1).

Finally we also found mutations in both RHO and PRPH2 genes that were not related to the splicing process: a stop loss in RP105^24^ and a missense mutation in RP135^25^, both in RHO, and a missense mutation in PRPH2 (p.Gly266Asp) in patient RP19S^26^. Patient RP19S was included in this study since he is the son of a patient with a mutation in PRPH2 that we had diagnosed previously^20^. Patient RP19S was asymptomatic at the initial diagnosis, when he was eight years old. However, two years later his molecular diagnosis confirmed the presence of the p.Gly266Asp mutation, therefore he was re-examined. This revealed a granular fundus and few bone spicules in the inferior periphery, with no signs of optical disc pallor or vascular attenuation. The visual field showed a concentric defect (preserving the central 18 degrees) with a hyperautofluorescent ring in the macula upon autofluorescence examination (see Supplementary Fig. S2). Additional family trees of the rest of the patients recruited in the present study are included in Supplementary Fig. S3.

## Discussion

In this work we have analysed the genotype and phenotype of a group of 29 adRP probands, using targeted NGS and Sanger sequencing to analyse 31 genes. We were able to detect putative disease-causing mutations in 14 out of the 29 probands analysed. This resulted in a clinically relevant genetic diagnosis ratio of 48.28%, which is comparable to values reported previously, ranging from about 24% to 88%^6^,^7^,^27^,^28^,^29^,^30^,^31^,^32^,^33^. Several factors may be responsible for this wide range of diagnosis ratios reported, including the approach used or the nature of the cohort involved. In the present study, part of our cohort of adRP patients was already diagnosed in a previous study in which we screened some of the most prevalent adRP genes^14^,^20^, therefore this might have contributed to the diagnostic ratio obtained.

Nevertheless there is still a missing fraction of about 51% unsolved cases among our adRP cohort of 29 patients. One possible explanation is the presence of mutations in regions outside the 31 genes analysed, such as deep intronic regions. Another possibility is the presence of changes not detected by our analysis due to limitations in the design of our panel of target genes, such as large genomic rearrangements and mutations in novel genes. As such, it seems that the combination of NGS with other technologies, such as Multiplex Ligation-dependent Probe Amplification (MLPA) or Comparative Genomic Hybridisation arrays (aCGH), will be needed in order to address those genomic aberrations caused by copy number variations (CNV). Another possible explanation is the presence of novel RP genes among our patients, since most of them belong to the Basque province of Gipuzkoa, a well-known genetically homogeneous region^34^. Consequently, sequencing of the whole exome/genome could help in the discovery of novel RP genes.

A remarkable finding was the high prevalence of mutations affecting the splicing process among our families (11 out of 29 probands studied), representing 38% of the probands in our adRP cohort.

Most mutations were the Ser1087Leu mutation found in SNRNP200. This gene encodes for the 200-kDa helicase hBrr2. During splicing, the spliceosome undergoes structural rearrangements that are mediated by several RNA helicases including hBrr2, which is essential for unwinding of the U4/U6 snRNP duplex, a key step in the catalytic activation of the spliceosome complex^35^,^36^. hBrr2 comprises two helicase modules, one active and the other with regulatory activity.

All six mutations identified in SNRNP200 to date, including the Ser1087Leu mutation, are located in the hBrr2 protein region containing the first DExD-helicase module, a key component for the U4-U6 unwinding function *in vivo* and *in vitro* and for cell survival^35^,^36^,^37^. The first of the two consecutive Hel308-like modules, which comprises a DExD/H domain and a Sec63 domain, shows the highest level of conservation among species, thus pointing to its functional relevance^38^. The Ser1087Leu mutation has been reported to reduce unwinding activity and to promote the use of cryptic splice sites, thus pointing to an influence of splicing fidelity^22^,^39^.

Although most cases (9/29) were due to mutations affecting genes SNRNP200 and PRPF8 that code for spliceosomal proteins, splice-site mutations in RHO were also detected (2/29). The percentage of adRP probands with mutations affecting either spliceosome core factors or the splice site of several adRP genes accounted for 5–14.5% of all cases of adRP in previous studies^4^,^7^,^40^,^41^. With regard to mutations in the SNRNP200 gene, although these were only initially described in two Chinese families^21^,^22^, they have since been reported to contribute to a significant portion of cases of adRP in the Caucasian population, ranging from 1.5% to 4.2%^4^,^40^,^42^,^43^.

The relatively high prevalence of splicing-related mutations found in our study is likely explained by the founder effect of two of the genes, which were present in very small and rather isolated Spanish populations.

Splicing modulation has been proposed as a therapeutic approach for several diseases. Two of the most advanced approaches in this regard are based on the use of modified antisense oligonucleotides (ASOs) to target specific RNA sequences and redirect splicing, and small molecules as modulators of the splicing process. A representative example of this approach is exon skipping for Duchenne muscular dystrophy (DMD), where the muscular protein dystrophin is prematurely truncated by mutations that disrupt the open reading frame, thus leading to a non-functional protein. Exon skipping creates an internally deleted and shorter than normal but partially functional protein, which leads to a much less severe phenotype in animal models of DMD. With respect to approaches based on small molecules and peptides, several splicing modulators have been shown to be effective in myotonic dystrophy (DM) and cancer^18^,^44^.

As regards retinal dystrophies, most advanced therapeutic approaches that target splicing are aimed at correcting the splicing of individual genes using mutation-adapted U1 small nuclear RNA for the RPGR gene^45^ or spliceosome-mediated RNA trans-splicing in RHO^46^. Both these approaches are based on cellular and animal models and have provided encouraging results. Once in the clinic, these promising approaches could be generalised and applied to other genes with splice donor site mutations^45^ and to all adRP genes rather than only to RPGR and RHO, respectively^46^.

With regard to therapeutic approaches targeting the splicing machinery, we are unaware of their use in retinal diseases. However, since the first steps towards the use of such therapeutic strategies have already been made for other diseases, it is plausible to imagine a broadening of the applications of small molecules to reverse aberrant splicing for other diseases, including retinal dystrophies, in the near future once our understanding of the mechanisms of the disease, and delivery systems and other technical issues, have been improved.

In summary, the combination of NGS with Sanger sequencing has allowed us to achieve a diagnostic rate of over 48%. As such, the methodology described herein exhibits a high diagnostic yield when applied to a well-defined adRP group and a relatively high number of genes. This will be of clinical relevance once ongoing studies on therapeutic options directed at manipulating splicing are completed.

## Materials and Methods

### Study subjects

RP patients were diagnosed at the Ophthalmology Department of Donostia University Hospital (San Sebastian, Spain). Diagnostic criteria were night blindness, peripheral visual field loss, pigmentary deposits resembling bone spicules, attenuation of retinal vessels, pallor of the optic disc and diminution in a- and b-wave amplitudes in the electroretinogram^47^. A total of 29 Spanish probands with a family tree compatible with adRP were included. Samples from an additional four patients, three corresponding to patients with known mutations that we had detected in previous analysis and one from a non-affected individual, were included as positive and negative controls, respectively^14^,^20^. Family trees were generated from information obtained from probands. All procedures performed in studies involving human participants received approval from the institutional research ethics committee and were in accordance with the Declaration of Helsinki (2013) or comparable ethical standards. Informed consent was obtained from all individual participants included in the study. For a detailed description of clinical features of all patients recruited in the present study see Supplementary Table S2.

### Human sample collection

High molecular weight DNA was extracted from blood samples from RP patients and their available family members. Total DNA from samples was extracted and isolated using an AutoGenFlex STAR instrument (AutoGen, Holliston, MA, USA) together with the FlexiGene DNA Kit (Qiagen, Hilden, Germany) following the manufacturer´s instructions. DNA concentrations were measured using a Nanodrop spectrophotometer (ND-1000, Thermo Scientific NanoDrop Products, Wilmington, DE, USA) an only those samples with 260/280 ratios ≥1.8 and 260/230 ratios ≥2 were used. DNA samples were stored at −80 °C.

### Amplicon Library preparation

A total of 663 primer pairs were designed and grouped in two Ion AmpliSeq Primer Pools to flank 31 IRD genes with a total coverage of 98.37% using the Ion AmpliSeq Designer software (www.ampliseq.com). The regions excluded by the design represented only 1.63% of the total. Although most of the genes were related to adRP, representative genes associated with dominant forms of Leber congenital amaurosis and cone-rod dystrophies were also included since the clinic symptoms associated with these genes are often hard to distinguish from those associated with RP (RetNet; https://sph.uth.edu/retnet/disease.htm) (see Supplementary Table S3). The Ion AmpliSeq Library Preparation Kit v2.0 (Life Technologies, Foster City, CA, USA) was used to construct an amplicon library from genomic target regions with a maximum read length of approximately 200 base pairs (average length, 142 bp) for shotgun sequencing on the PGM. Briefly, target genomic regions were amplified by simple PCR using Ion AmpliSeq Primer Pools and 10 ng of each genomic DNA samples.

### Sequencing Analysis

#### Ion Torrent Personal Genome Machine (PGM)

NGS was carried out on a PGM following the Ion PGM 200 Sequencing Kit protocol. Briefly, enriched Ion Sphere particles (ISPs) were annealed with the Ion Sequencing primer and mixed with the PGM200 Sequencing Polymerase. The polymerase-bound and primer-activated ISPs were then loaded into the previously checked and washed Ion 316 Chips (Life Technologies) and, after selecting the run plan on the Ion PGM System software, these chips were subjected to 500 cycles of sequencing with the standard nucleotide flow order. Signal processing and base calling for the data generated during the PGM runs were performed using the Ion Torrent platform-specific analysis software Torrent Suite version 4.0 to generate sequence reads. The sequences generated were aligned to the GRCh37/hg19 human genome for detection of genomic variants in the sequenced samples.

#### Sanger sequencing

Sanger sequencing was used to confirm those mutations detected by NGS and for co-segregation analysis. Primers were designed at least 60 bp upstream and downstream of the mutation. The amplicons were purified after PCR amplification, (ExoSAP-IT, USB Corporation). Sequencing was performed by dye termination DNA reaction on a 16-capillary ABI 3130xl platform (Applied Biosystems) according to the manufacturer’s protocol. Sequences were analysed and compared with wild-type samples and reference sequences using the BioEdit Sequence Alignment Editor (Windows) and Ensembl and NCBI databases.

#### High resolution melting (HRM) analysis

HRM analysis was used to re-analyse those genomic regions with no or very low coverage in NGS platforms, following the previously described methodology^20^.

### Relevant variant identification and pathogenicity score

In order to determine genomic variants of relevance, we selected putative disease-causing variants using the following criteria: 1) variants previously reported as pathogenic, or 2) loss-of-function variants, such as stop gain, frameshift, small deletions or duplications (INDELS) and splice site variants, or 3) novel missense variants predicted to be damaging or highly pathogenic in at least four out of five web-based pathogenicity predictors, namely SIFT (<0.05), Polyphen2 (>0.750); PROVEAN^48^; GVGD^49^; MutationTaster^50^. Furthermore, all variants selected had to fulfil the criteria of having a Minor Allele Frequency (MAF) of less than 0.002, as obtained from human genome databases (see below), and being absent from Spanish in-house allele database with information from 578 unrelated Spanish individuals none of whom exhibited any IRD-related disease^51^ (http://csvs.babelomics.org/; see Supplementary Fig. S4).

## Additional Information

**How to cite this article**: Ezquerra-Inchausti, M. *et al*. High prevalence of mutations affecting the splicing process in a Spanish cohort with autosomal dominant retinitis pigmentosa. *Sci. Rep.* **7**, 39652; doi: 10.1038/srep39652 (2017).

**Publisher's note:** Springer Nature remains neutral with regard to jurisdictional claims in published maps and institutional affiliations.

## Supplementary Material

Supplementary Information

## Figures and Tables

**Table 1:** Summary of mutations responsible for Retinitis Pigmentosa.

Family	Gene	Mutation	Type	Ref	HSF	Prov	Sift	Ph	Mut TASTER
RP19S	PRPH2	NM_000322c.797G > A p.Gly266Asp	missense	26		D	0	0.99	Disease causing (0.999)
RP22RP37RP64RP101RP102RP134RP157	SNRNP200	NM_014014c.3260C > T p.Ser1087Leu	missense	21, 22		D	0	1	Disease causing (0.999)
RP90	PRPF8	NM_006445c.6974_6994del p.Val2325_Glu2330del	deletion	novel			n/a	n/a	Disease causing (0.999)
RP113	PRPF8	NM_006445c.6945delG p.Leu2315 Leufs*2336 Aspext*21	frameshift	novel			n/a	n/a	Disease causing (1)
RP133RP146	RHO	NM_000539c.937-1G>T	splice acceptor variant	23	Decrease 5′ acceptor site of exon 5 (90.7>61.75)		n/a	n/a	Disease causing (1)
RP105	RHO	NM_000539c.1045T>C p.Ter349Glu	stop loss	24			n/a	n/a	Polymorphism (0.999)
RP135	RHO	NM_000539c.568G>A p.Asp190Asn	missense	25		D	0	0.431	Disease causing (0.999)

Abbreviations: D: deleterious; HSF: human splicing finder; MUT TASTER: Mutation Taster; n/a: not available; PH: Polyphen; PROV: Provean; REF: bibliographic reference.

All variants were absent in a Spanish in-house allele database containing information from 578 unrelated Spanish individuals (Spanish controls). See *Materials and Methods* section for detailed information.

## References

[b1] Hartong D. T., Berson E. L. & Dryja T. P. Retinitis pigmentosa. Lancet 368, 1795–1809, doi: S0140-6736(06)69740-7 [pii]10.1016/S0140-6736(06)69740-7 (2006). 17113430 10.1016/S0140-6736(06)69740-7

[b2] Dryja T. P., Hahn L. B., Kajiwara K. & Berson E. L. Dominant and digenic mutations in the peripherin/RDS and ROM1 genes in retinitis pigmentosa. Invest. Ophthalmol. Vis. Sci. 38, 1972–1982 (1997). 9331261

[b3] Mansergh F. C. . Retinitis pigmentosa and progressive sensorineural hearing loss caused by a C12258A mutation in the mitochondrial MTTS2 gene. Am. J. Hum. Genet. 64, 971–985 (1999). 10090882 10.1086/302344 PMC1377821

[b4] Daiger S. P., Bowne S. J. & Sullivan L. S. Genes and Mutations Causing Autosomal Dominant Retinitis Pigmentosa. Cold Spring Harb. Perspect. Med. 5, doi: 10.1101/cshperspect.a017129 (2014). PMC4588133 25304133

[b5] Anasagasti A., Irigoyen C., Barandika O., Lopez de Munain A. & Ruiz-Ederra J. Current mutation discovery approaches in Retinitis Pigmentosa. Vision Res. 75, 117–129, doi: S0042-6989(12)00303-3 [pii]10.1016/j.visres.2012.09.012 (2012). 23022136 10.1016/j.visres.2012.09.012

[b6] Daiger S. P. . Application of Next-Generation Sequencing to Identify Genes and Mutations Causing Autosomal Dominant Retinitis Pigmentosa (adRP). Advances in experimental medicine and biology 801, 123–129, doi: 10.1007/978-1-4614-3209-8_16 (2014). 24664689 PMC4121110

[b7] Fernandez-San Jose P. . Targeted Next-Generation Sequencing Improves the Diagnosis of Autosomal Dominant Retinitis Pigmentosa in Spanish Patients. Invest. Ophthalmol. Vis. Sci. 56, 2173–2182, doi: 10.1167/iovs.14-16178 (2015). 25698705

[b8] Wang F. . A missense mutation in HK1 leads to autosomal dominant retinitis pigmentosa. Invest. Ophthalmol. Vis. Sci. 55, 7159–7164, doi: 10.1167/iovs.14-15520 (2014). 25316723 PMC4224578

[b9] Ma X. . Whole-exome sequencing identifies OR2W3 mutation as a cause of autosomal dominant retinitis pigmentosa. Scientific reports 5, 9236, doi: 10.1038/srep09236 (2015). 25783483 PMC4363838

[b10] Liu Y. . SPP2 Mutations Cause Autosomal Dominant Retinitis Pigmentosa. Scientific reports 5, 14867, doi: 10.1038/srep14867 (2015). 26459573 PMC4602186

[b11] Chen X. . PRPF4 mutations cause autosomal dominant retinitis pigmentosa. Hum. Mol. Genet. 23, 2926–2939, doi: 10.1093/hmg/ddu005 (2014). 24419317

[b12] Dewey F. E. . Clinical interpretation and implications of whole-genome sequencing. JAMA: the journal of the American Medical Association 311, 1035–1045, doi: 10.1001/jama.2014.1717 (2014). 24618965 PMC4119063

[b13] Wahl M. C., Will C. L. & Luhrmann R. The spliceosome: design principles of a dynamic RNP machine. Cell 136, 701–718, doi: 10.1016/j.cell.2009.02.009 (2009). 19239890

[b14] Barandika O. . A Cost-Effective Mutation Screening Strategy for Inherited Retinal Dystrophies. Ophthalmic research, doi: 10.1159/000445690 (2016). 27160245

[b15] Havens M. A., Duelli D. M. & Hastings M. L. Targeting RNA splicing for disease therapy. Wiley interdisciplinary reviews. RNA 4, 247–266, doi: 10.1002/wrna.1158 (2013). 23512601 PMC3631270

[b16] Liu M. M. & Zack D. J. Alternative splicing and retinal degeneration. Clin. Genet. 84, 142–149, doi: 10.1111/cge.12181 (2013). 23647439 PMC4147722

[b17] Scotti M. M. & Swanson M. S. RNA mis-splicing in disease. Nat. Rev. Genet. 17, 19–32, doi: 10.1038/nrg.2015.3 (2016). 26593421 PMC5993438

[b18] Singh R. K. & Cooper T. A. Pre-mRNA splicing in disease and therapeutics. Trends in molecular medicine 18, 472–482, doi: 10.1016/j.molmed.2012.06.006 (2012). 22819011 PMC3411911

[b19] Cao H. . Temporal and tissue specific regulation of RP-associated splicing factor genes PRPF3, PRPF31 and PRPC8–implications in the pathogenesis of RP. PLoS One 6, e15860, doi: 10.1371/journal.pone.0015860 (2011). 21283520 PMC3023711

[b20] Anasagasti A. . Genetic high throughput screening in Retinitis Pigmentosa based on high resolution melting (HRM) analysis. Experimental eye research 116, 386–394 (2013). 24416769 10.1016/j.exer.2013.10.011

[b21] Zhao C. . A novel locus (RP33) for autosomal dominant retinitis pigmentosa mapping to chromosomal region 2cen-q12.1. Hum. Genet. 119, 617–623, doi: 10.1007/s00439-006-0168-3 (2006). 16612614

[b22] Zhao C. . Autosomal-dominant retinitis pigmentosa caused by a mutation in SNRNP200, a gene required for unwinding of U4/U6 snRNAs. Am. J. Hum. Genet. 85, 617–627, doi: 10.1016/j.ajhg.2009.09.020 (2009). 19878916 PMC2775825

[b23] Bell C., Converse C. A., Hammer H. M., Osborne A. & Haites N. E. Rhodopsin mutations in a Scottish retinitis pigmentosa population, including a novel splice site mutation in intron four. The British journal of ophthalmology 78, 933–938 (1994). 7819178 10.1136/bjo.78.12.933 PMC504996

[b24] Hollingsworth T. J. & Gross A. K. The severe autosomal dominant retinitis pigmentosa rhodopsin mutant Ter349Glu mislocalizes and induces rapid rod cell death. The Journal of biological chemistry 288, 29047–29055, doi: 10.1074/jbc.M113.495184 (2013). 23940033 PMC3790004

[b25] Keen T. J. . Autosomal dominant retinitis pigmentosa: four new mutations in rhodopsin, one of them in the retinal attachment site. Genomics 11, 199–205 (1991). 1765377 10.1016/0888-7543(91)90119-y

[b26] Sohocki M. M. . Prevalence of mutations causing retinitis pigmentosa and other inherited retinopathies. Hum. Mutat. 17, 42–51, doi: 10.1002/1098-1004(2001)17:1<42::AID-HUMU5>3.0.CO;2-K[pii]10.1002/1098-1004(2001)17:1<42::AID-HUMU5>3.0.CO;2-K(2001). PMC2585107 11139241

[b27] Bowne S. J. . Identification of disease-causing mutations in autosomal dominant retinitis pigmentosa (adRP) using next-generation DNA sequencing. Invest. Ophthalmol. Vis. Sci. 52, 494–503, doi: 10.1167/iovs.10-6180 (2011). 20861475 PMC3053293

[b28] Audo I. . Development and application of a next-generation-sequencing (NGS) approach to detect known and novel gene defects underlying retinal diseases. Orphanet J. Rare Dis. 7, 8, doi: 10.1186/1750-1172-7-8 (2012). 22277662 PMC3352121

[b29] Sullivan L. S. . A dominant mutation in hexokinase 1 (HK1) causes retinitis pigmentosa. Invest. Ophthalmol. Vis. Sci. 55, 7147–7158, doi: 10.1167/iovs.14-15419 (2014). 25190649 PMC4224580

[b30] Eisenberger T. . Increasing the yield in targeted next-generation sequencing by implicating CNV analysis, non-coding exons and the overall variant load: the example of retinal dystrophies. PLoS One 8, e78496, doi: 10.1371/journal.pone.0078496 (2013). 24265693 PMC3827063

[b31] Glockle N. . Panel-based next generation sequencing as a reliable and efficient technique to detect mutations in unselected patients with retinal dystrophies. Eur. J. Hum. Genet. 22, 99–104, doi: 10.1038/ejhg.2013.72 (2014). 23591405 PMC3865404

[b32] Oishi M. . Comprehensive molecular diagnosis of a large cohort of Japanese retinitis pigmentosa and Usher syndrome patients by next-generation sequencing. Invest. Ophthalmol. Vis. Sci. 55, 7369–7375, doi: 10.1167/iovs.14-15458 (2014). 25324289

[b33] Gonzalez-del Pozo M. . Mutation screening of multiple genes in Spanish patients with autosomal recessive retinitis pigmentosa by targeted resequencing. PLoS One 6, e27894, doi: 10.1371/journal.pone.0027894PONE-D-11-14335 [pii] (2011). 22164218 PMC3229495

[b34] Champe S. P., Rao P. & Chang A. An endogenous inducer of sexual development in Aspergillus nidulans. Journal of general microbiology 133, 1383–1387, doi: 10.1099/00221287-133-5-1383 (1987). 3309182

[b35] Raghunathan P. L. & Guthrie C. RNA unwinding in U4/U6 snRNPs requires ATP hydrolysis and the DEIH-box splicing factor Brr2. Curr. Biol. 8, 847–855 (1998). 9705931 10.1016/s0960-9822(07)00345-4

[b36] Ruzickova S. & Stanek D. Mutations in spliceosomal proteins and retina degeneration. RNA Biol. 1–9, doi: 10.1080/15476286.2016.1191735 (2016). PMC5449078 27302685

[b37] Kim D. H. & Rossi J. J. The first ATPase domain of the yeast 246-kDa protein is required for *in vivo* unwinding of the U4/U6 duplex. RNA 5, 959–971 (1999). 10411139 10.1017/s135583829999012x PMC1369820

[b38] Zhang L. . Structural evidence for consecutive Hel308-like modules in the spliceosomal ATPase Brr2. Nat. Struct. Mol. Biol. 16, 731–739, doi: 10.1038/nsmb.1625 (2009). 19525970 PMC2743687

[b39] Cvackova Z., Mateju D. & Stanek D. Retinitis pigmentosa mutations of SNRNP200 enhance cryptic splice-site recognition. Hum. Mutat. 35, 308–317, doi: 10.1002/humu.22481 (2014). 24302620

[b40] Coussa R. G. . Genotype and Phenotype Studies in Autosomal Dominant Retinitis Pigmentosa (adRP) of the French Canadian Founder Population. Invest. Ophthalmol. Vis. Sci. 56, 8297–8305, doi: 10.1167/iovs.15-17104 (2015). 26720483 PMC4699406

[b41] Sullivan L. S. . Prevalence of disease-causing mutations in families with autosomal dominant retinitis pigmentosa: a screen of known genes in 200 families. Invest. Ophthalmol. Vis. Sci. 47, 3052–3064, doi: 10.1167/iovs.05-1443 (2006). 16799052 PMC2585061

[b42] Benaglio P. . Next generation sequencing of pooled samples reveals new SNRNP200 mutations associated with retinitis pigmentosa. Hum. Mutat. 32, E2246–2258, doi: 10.1002/humu.21485 (2011). 21618346

[b43] Bowne S. J. . Mutations in the small nuclear riboprotein 200 kDa gene (SNRNP200) cause 1.6% of autosomal dominant retinitis pigmentosa. Mol. Vis. 19, 2407–2417 (2013). 24319334 PMC3850977

[b44] Nakajima H. . New antitumor substances, FR901463, FR901464 and FR901465. I. Taxonomy, fermentation, isolation, physico-chemical properties and biological activities. The Journal of antibiotics 49, 1196–1203 (1996). 9031664 10.7164/antibiotics.49.1196

[b45] Glaus E., Schmid F., Da Costa R., Berger W. & Neidhardt J. Gene therapeutic approach using mutation-adapted U1 snRNA to correct a RPGR splice defect in patient-derived cells. Molecular therapy: the journal of the American Society of Gene Therapy 19, 936–941, doi: 10.1038/mt.2011.7 (2011). 21326217 PMC3098652

[b46] Berger A. . Repair of rhodopsin mRNA by spliceosome-mediated RNA trans-splicing: a new approach for autosomal dominant retinitis pigmentosa. Molecular therapy: the journal of the American Society of Gene Therapy 23, 918–930, doi: 10.1038/mt.2015.11 (2015). 25619725 PMC4427870

[b47] Hamel C. Retinitis pigmentosa. Orphanet J. Rare. Dis. 1, 40, doi: 1750-1172-1-40 [pii]10.1186/1750-1172-1-40 (2006). 17032466 10.1186/1750-1172-1-40 PMC1621055

[b48] Choi Y., Sims G. E., Murphy S., Miller J. R. & Chan A. P. Predicting the functional effect of amino acid substitutions and indels. PLoS One 7, e46688, doi: 10.1371/journal.pone.0046688 (2012). 23056405 PMC3466303

[b49] Mathe E. . Computational approaches for predicting the biological effect of p53 missense mutations: a comparison of three sequence analysis based methods. Nucleic acids research 34, 1317–1325, doi: 10.1093/nar/gkj518 (2006). 16522644 PMC1390679

[b50] Schwarz J. M., Rodelsperger C., Schuelke M. & Seelow D. MutationTaster evaluates disease-causing potential of sequence alterations. Nature methods 7, 575–576, doi: 10.1038/nmeth0810-575 (2010). 20676075

[b51] Alonso R. . Babelomics 5.0: functional interpretation for new generations of genomic data. Nucleic acids research 43, W117–121, doi: 10.1093/nar/gkv384 (2015). 25897133 PMC4489263

