# Supplementary material for: High prevalence of mutations affecting the splicing process in a Spanish cohort with autosomal dominant retinitis pigmentosa
Source: Sci Rep. 2017 Jan 3;7:39652. doi: 10.1038/srep39652 (PMC5206707; doi:10.1038/srep39652)
Supplement: Supplementary Information [file srep39652-s1.pdf]

Supplementary Information

High prevalence of mutations affecting the splicing process in a Spanish cohort with autosomal dominant retinitis pigmentosa

Maitane Ezquerra-Inchausti [1¶], Olatz Barandika[1¶], Ander Anasagasti[1], Cristina Irigoyen[1,2], Adolfo López de Munain[1,3,4,5], Javier Ruiz-Ederra[1*].

[1] Division of Neurosciences, Biodonostia Health Research Institute, San Sebastián, Spain; [2]Department of Ophthalmology, Donostia University Hospital, San Sebastián, Spain; [3]Department of Neurology, Donostia University Hospital, San Sebastián, Spain; [4]CIBERNED, Center for Networked Biomedical Research on Neurodegenerative Diseases, Institute of Health Carlos III, Ministry of Economy and Competitiveness, Spain; [5]Department of Neurosciences, University of the Basque Country UPV-EHU, Spain.

¶ These authors contributed equally to this work

* Correspondence: Javier Ruiz Ederra: Javier Ruiz-Ederra, PhD, Department of Neurosciences, Biodonostia Health Research Institute, Paseo Dr. Begiristain s/n, E-20014-San Sebastián, SPAIN.

Email: javiruizederra@yahoo.es/javier.ruizederra@osakidetza.eus. Phone: (+34) 943-006128; Fax: (+34) 943006250

Supplementary Figure S1

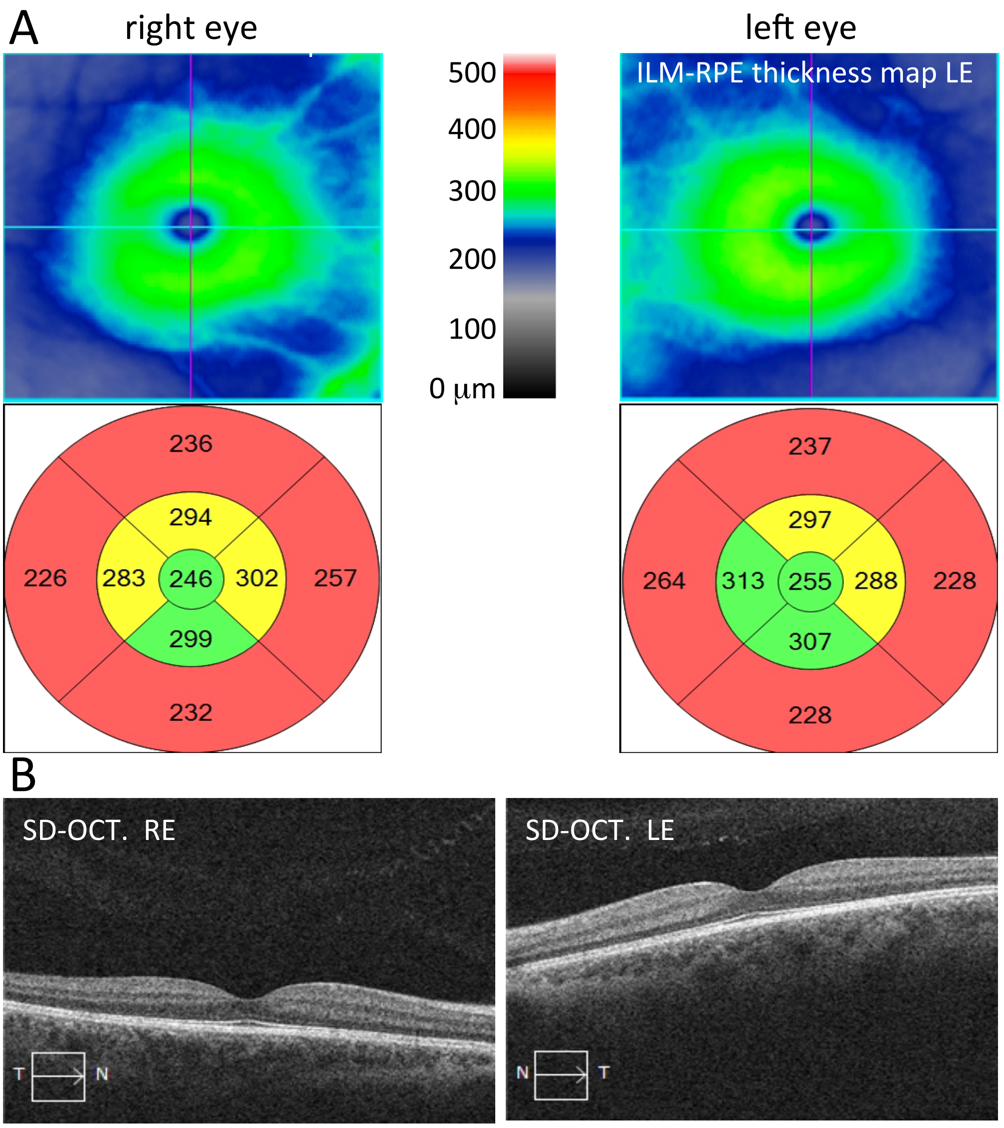

Supplementary Figure S1. Optical coherence tomography (OCT). The c.937-1G>T mutation in RHO was found in a 21 year-old patient (indicated by an arrowhead in family tree in Figure 2C). , prior to clinical diagnosis. Visual fields, funduscopy and autofluorescence were normal. On OCT we can see what could be an early sign of RP: the thinning of the macula at the 6mm ring (red colour) (**A**). No disruption of the external limiting membrane or the photoreceptor layer was observed. No macular oedema or epiretinal membrane were seen in the OCT (**B**). Abbreviations: ILM-RPE: inner limiting membrane-retinal pigment epithelium; LE: left eye; RE: right eye; SD-OCT: spectral domain optical coherence tomography.

Supplementary Figure S2

A

B

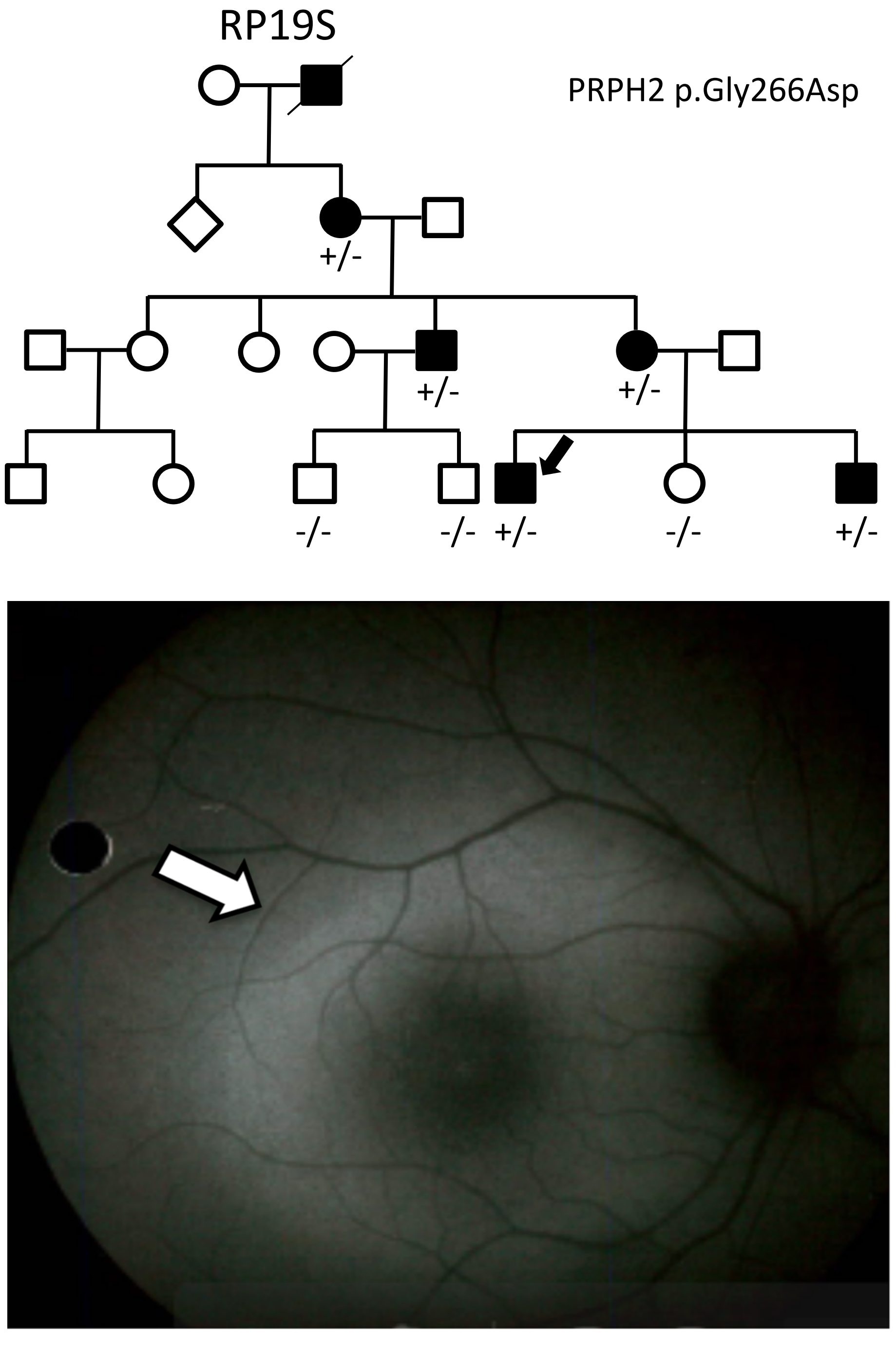

Supplementary Figure S2. Autofluorescence retinography and family tree for proband RP19S. Family tree. Genotypes are annotated as +/- (heterozygote); or -/- (wild type). Arrow indicate proband (**A**). Autofluorescence examination of the eye fundus shows a hyperautofluorescent ring in the macula (open arrow) (**B**).

Supplementary Figure S3

RP113

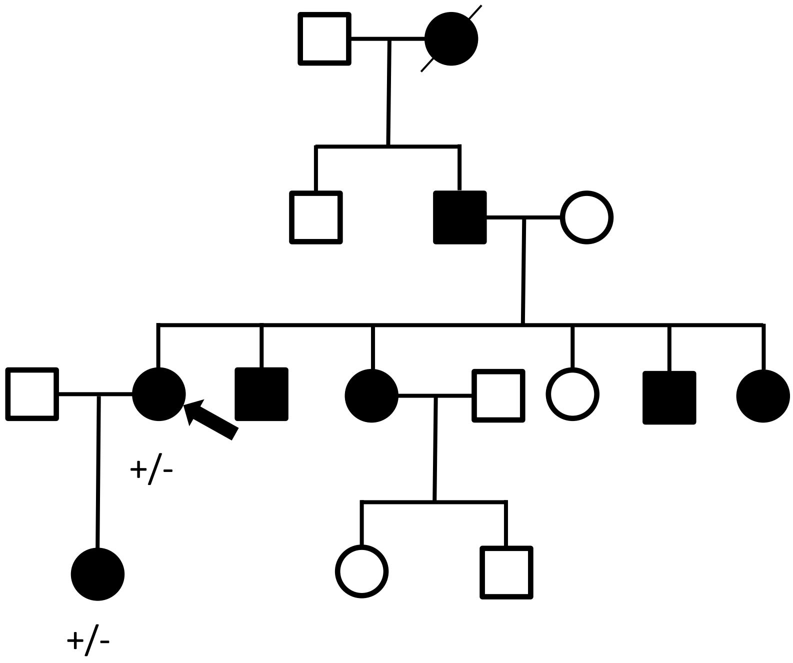

PRPF8 p.Leu2315Leufs*2336Aspext*21

RP90

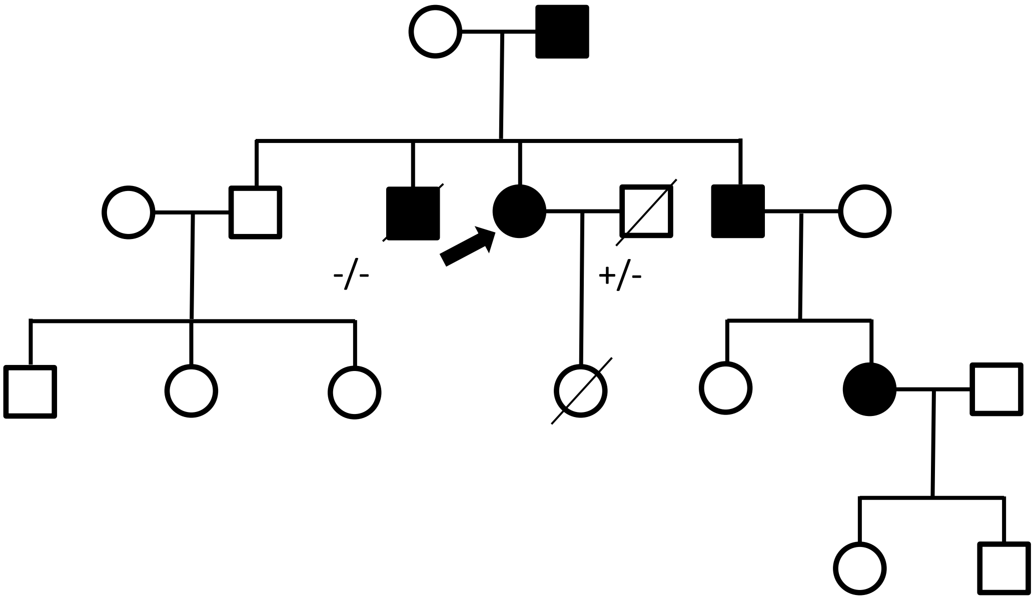

PRPF8 p.Val2325_Glu2330del

Supplementary Figure S3 (continued)

RP22

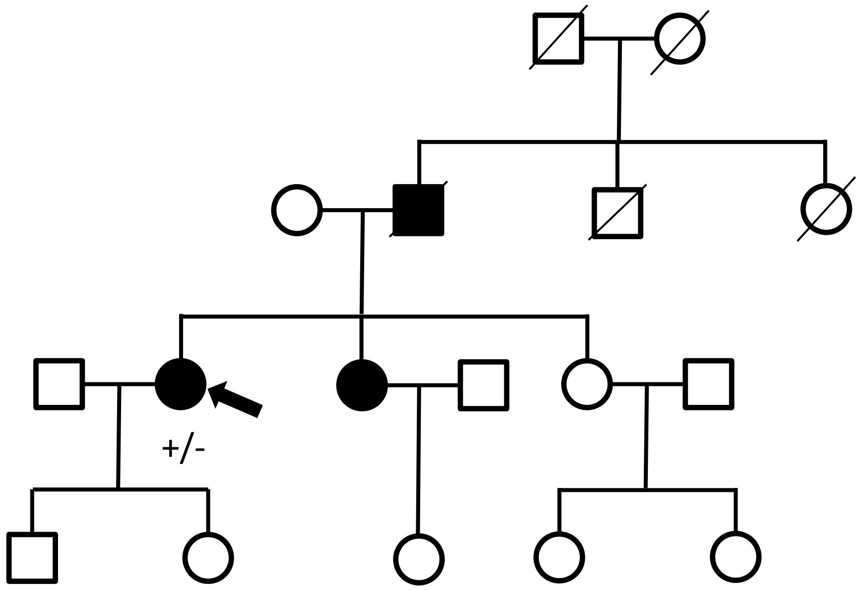

SNRNP200 p.Ser1087Leu

RP37

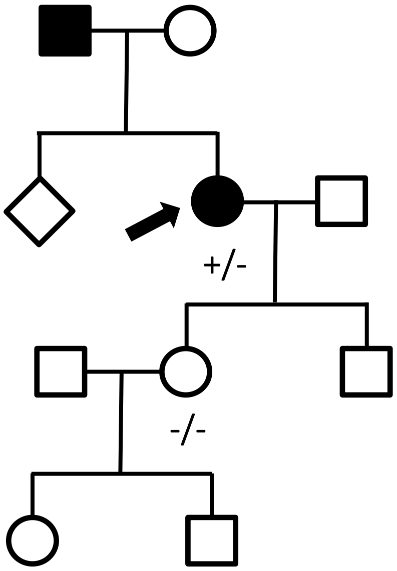

SNRNP200 p.Ser1087Leu

RP105

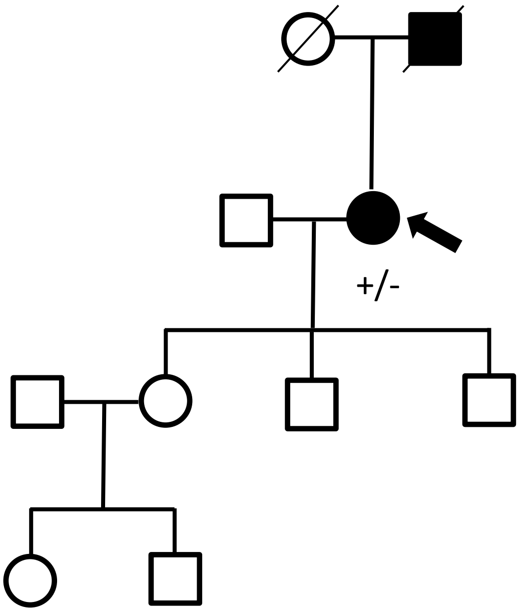

RHO p.Ter349Glu

Supplementary Figure S3 (continued)

RP101

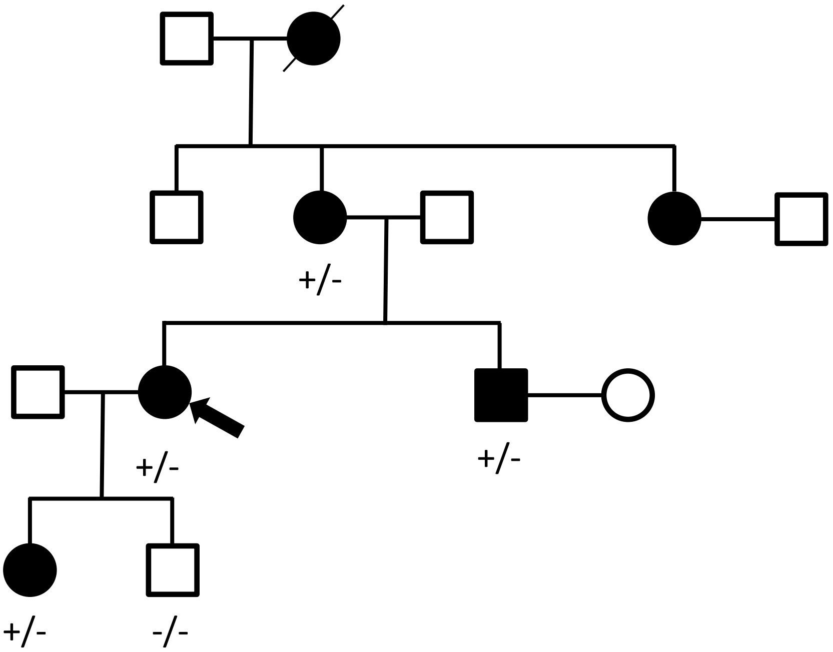

SNRNP200 p.Ser1087Leu

RP146

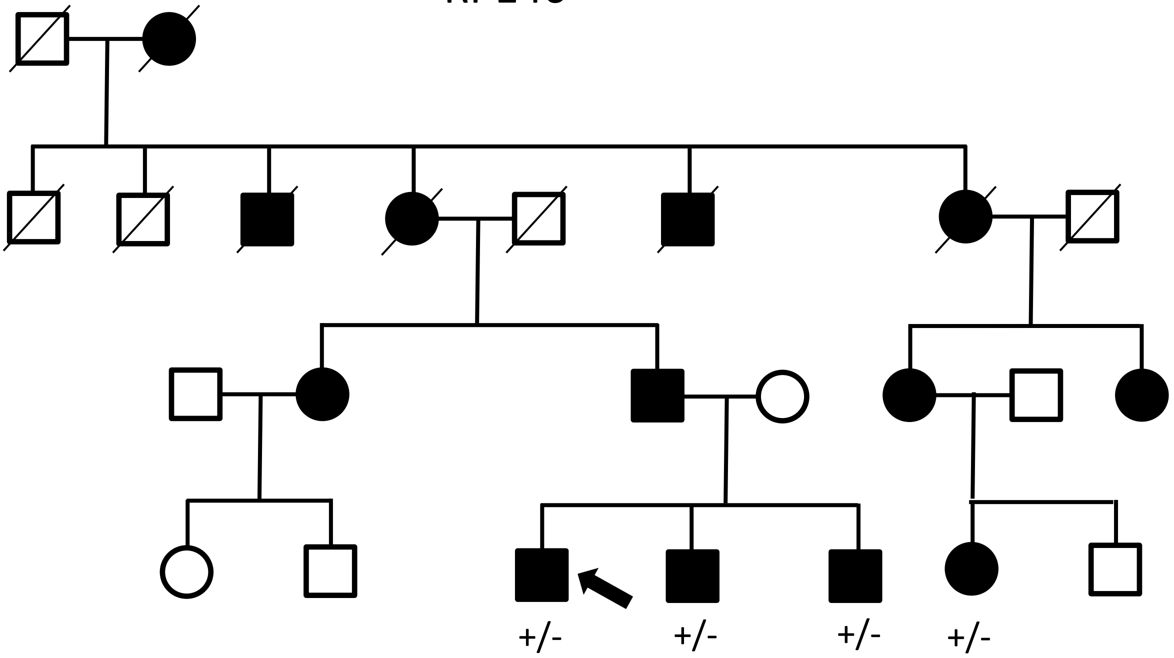

RHO c.937-1G>T

Supplementary Figure S3 (continued)

RP157

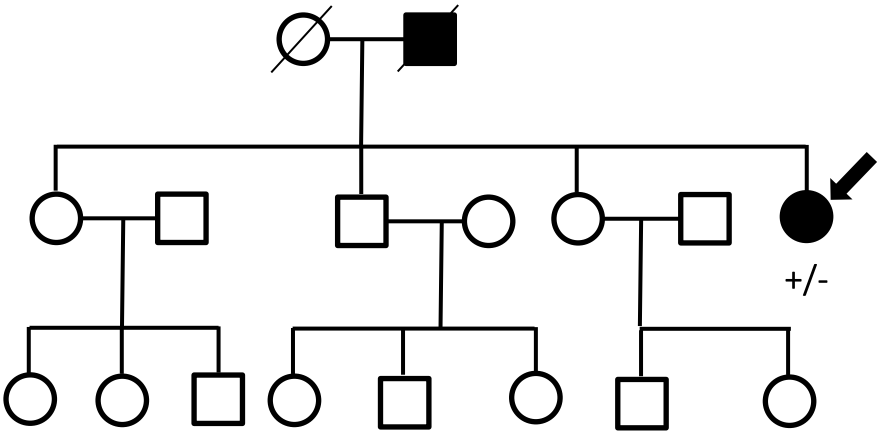

RP134

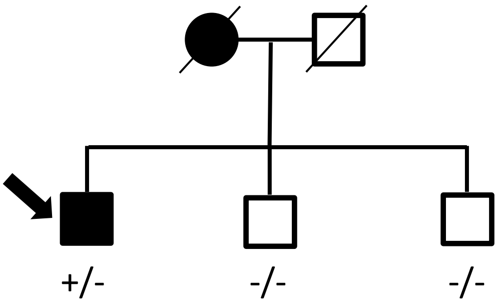

RP135

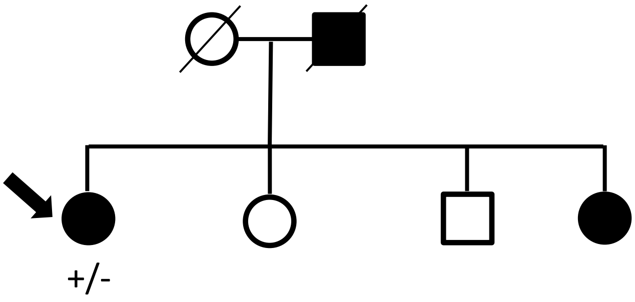

Supplementary Figure S3 (continued)

RP16

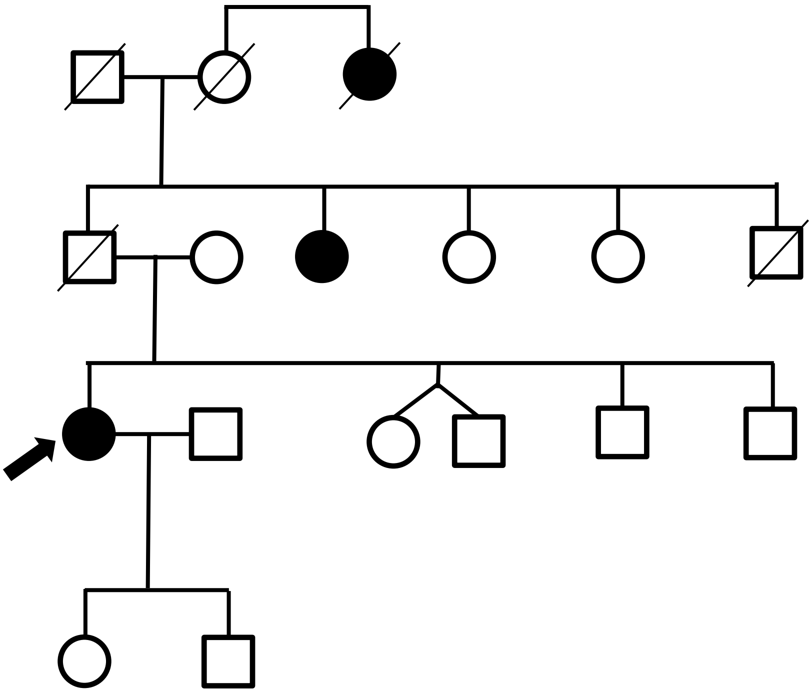

RP20

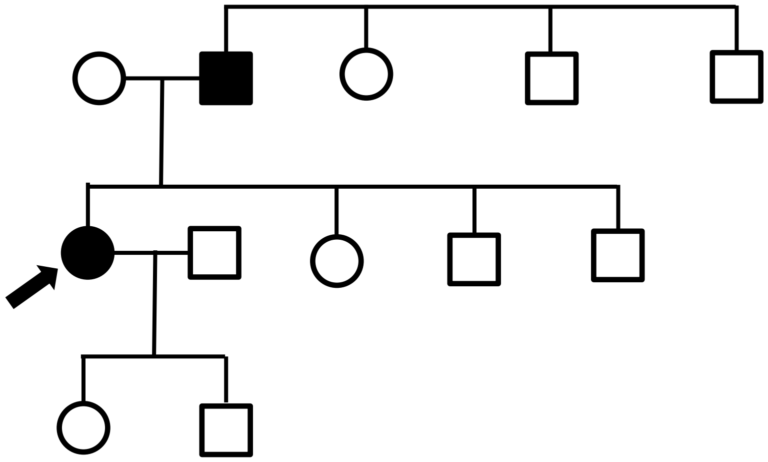

Supplementary Figure S3 (continued)

RP42

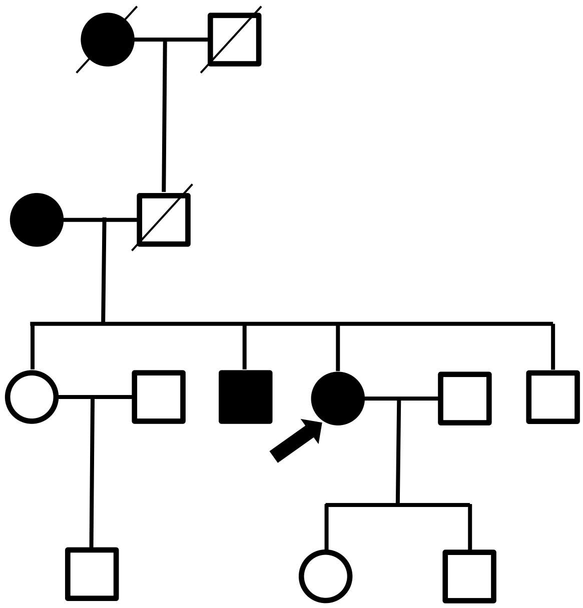

RP39

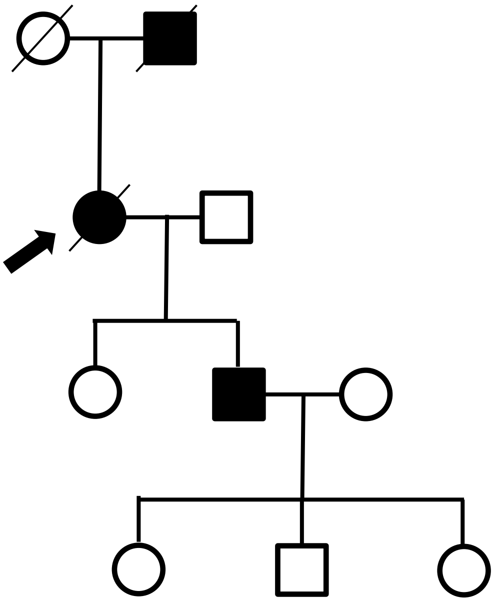

RP70

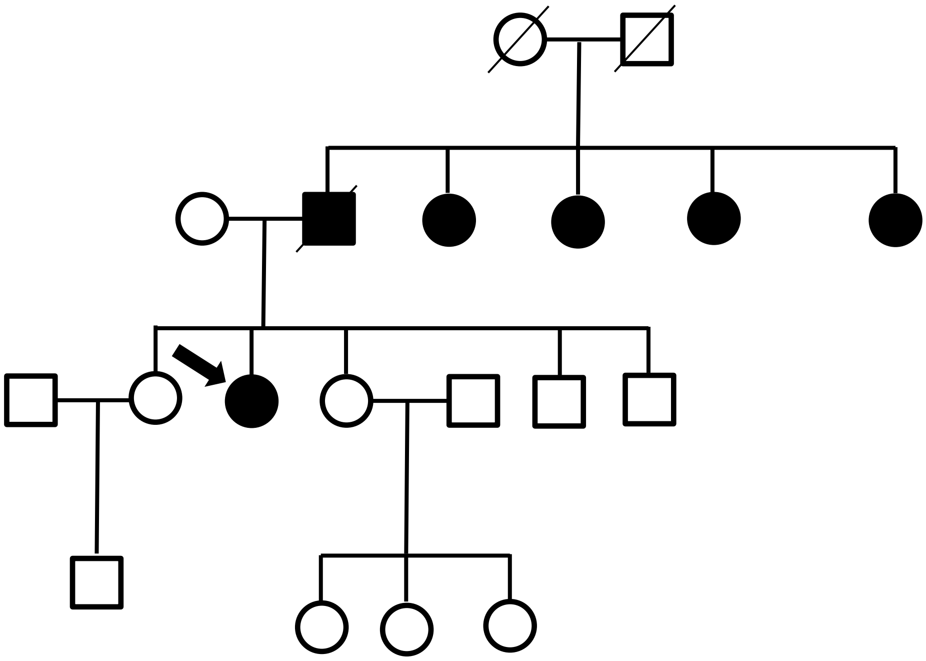

Supplementary Figure S3 (continued)

RP43

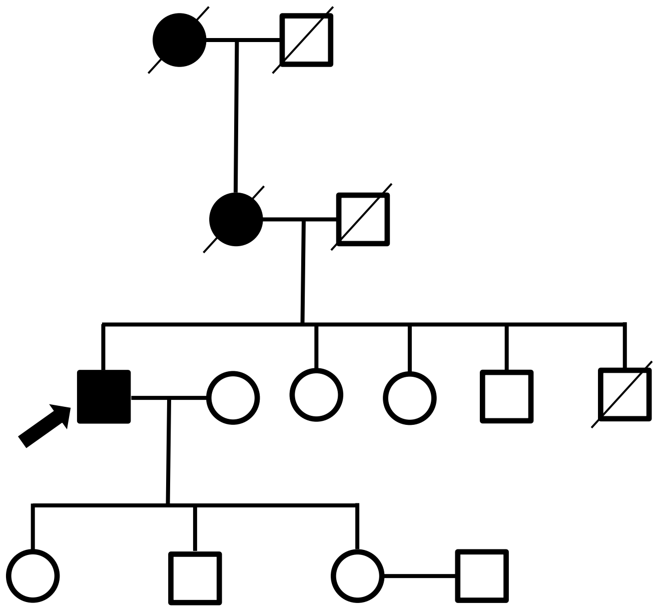

RP48

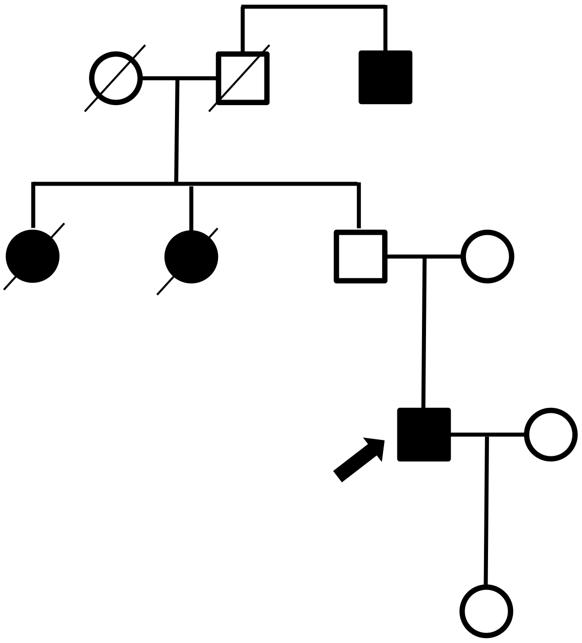

Supplementary Figure S3 (continued)

RP148

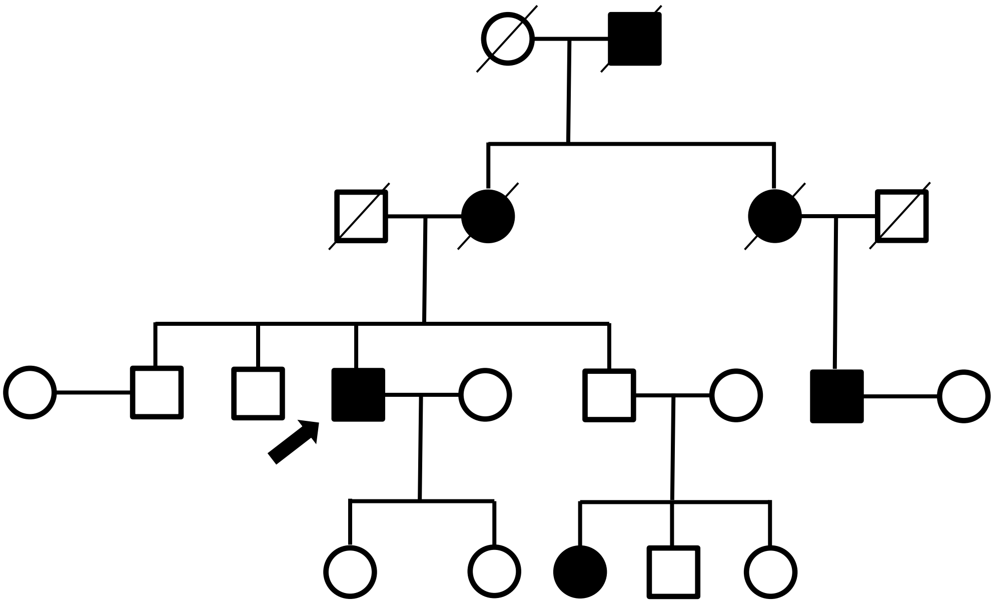

RP66

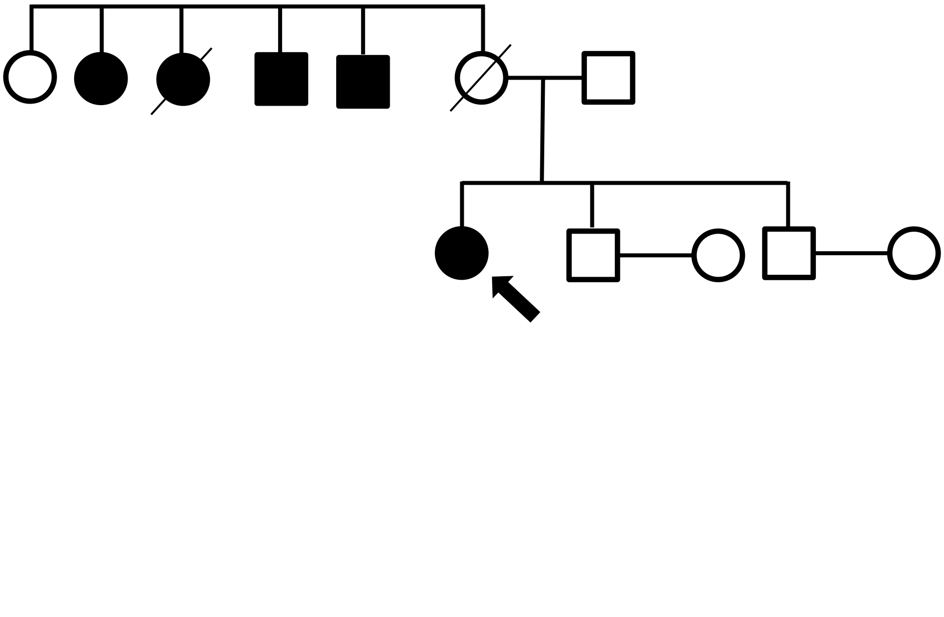

Supplementary Figure S3 (continued)

RP69

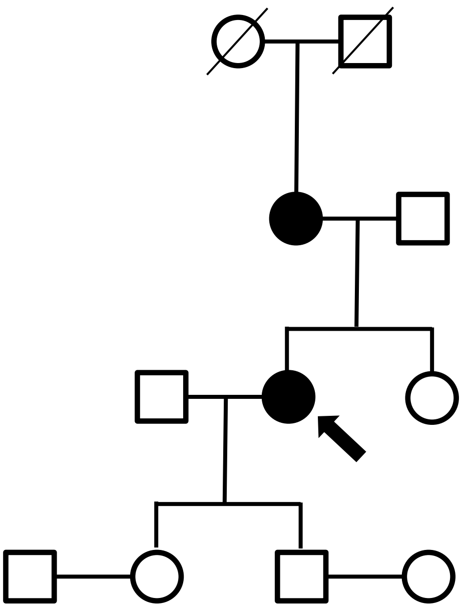

RP79

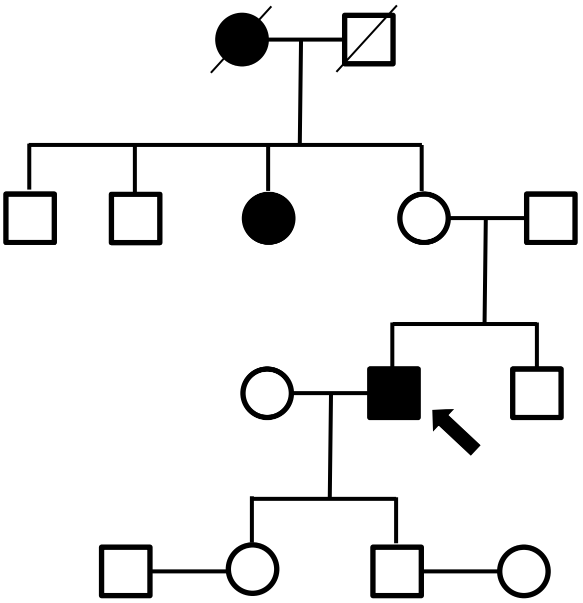

RP80

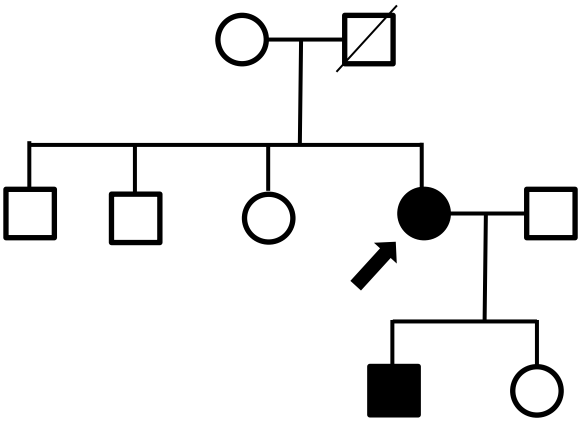

RP85

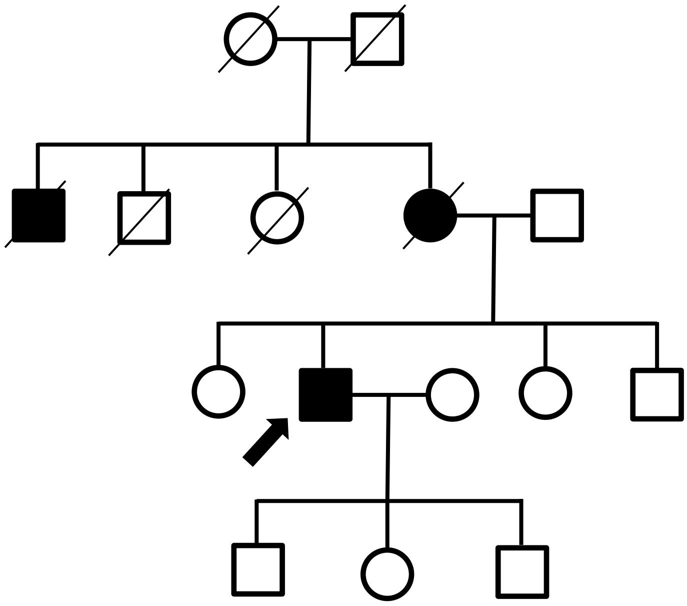

Supplementary Figure S3 (continued)

RP99

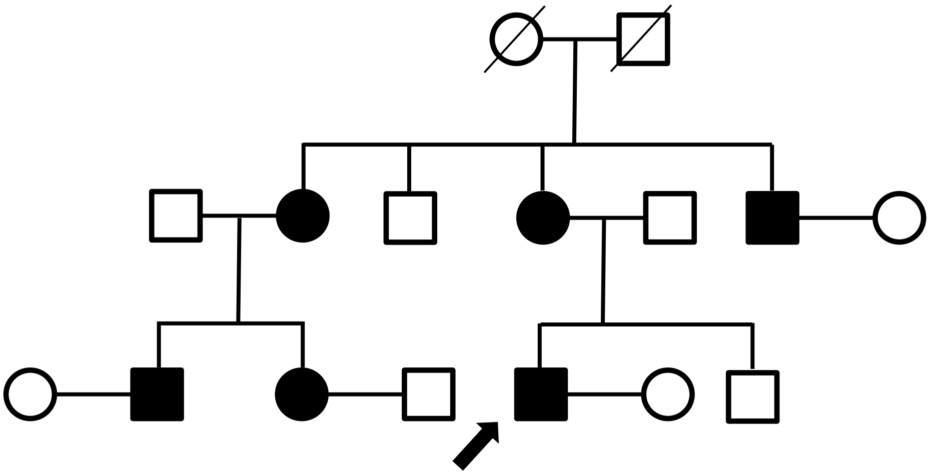

RP2

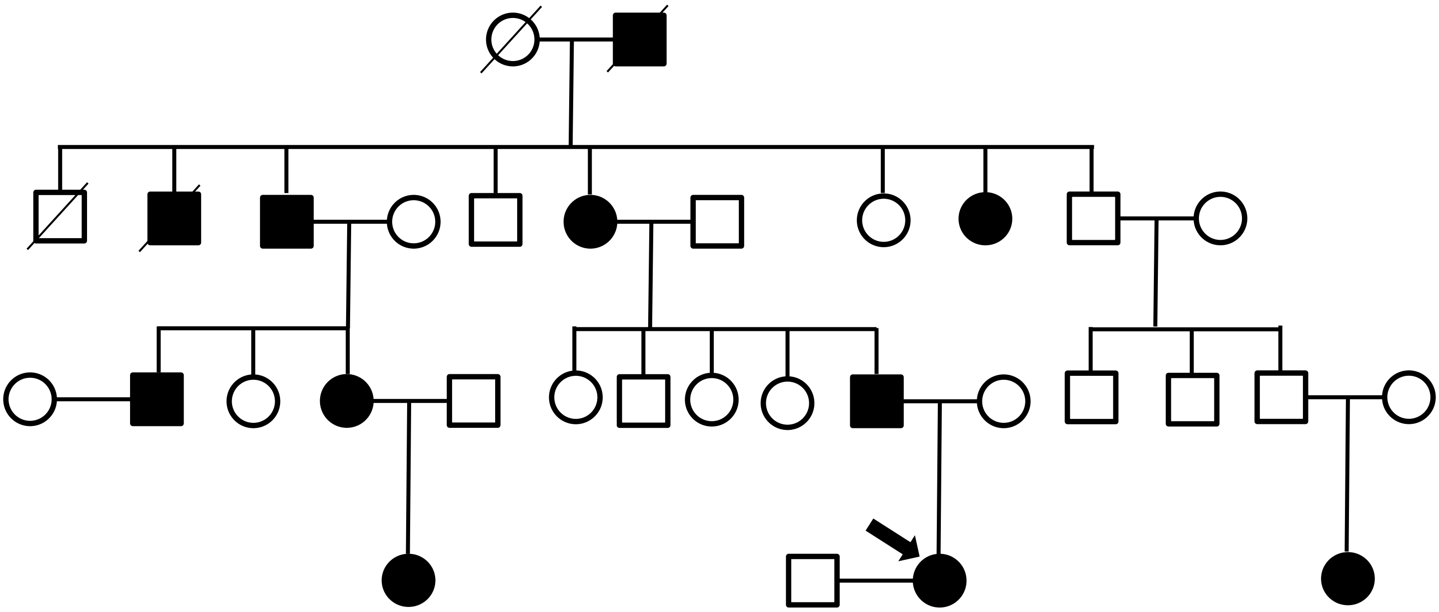

Supplementary Figure S3. Family trees for all probands recruited in the study, excluding those described in Figure 2 and Sup. Fig. 1. Genotypes of those cases diagnosed are annotated as +/- (heterozygote); or -/- (wild type). Arrow indicate proband.

Supplementary Figure S4

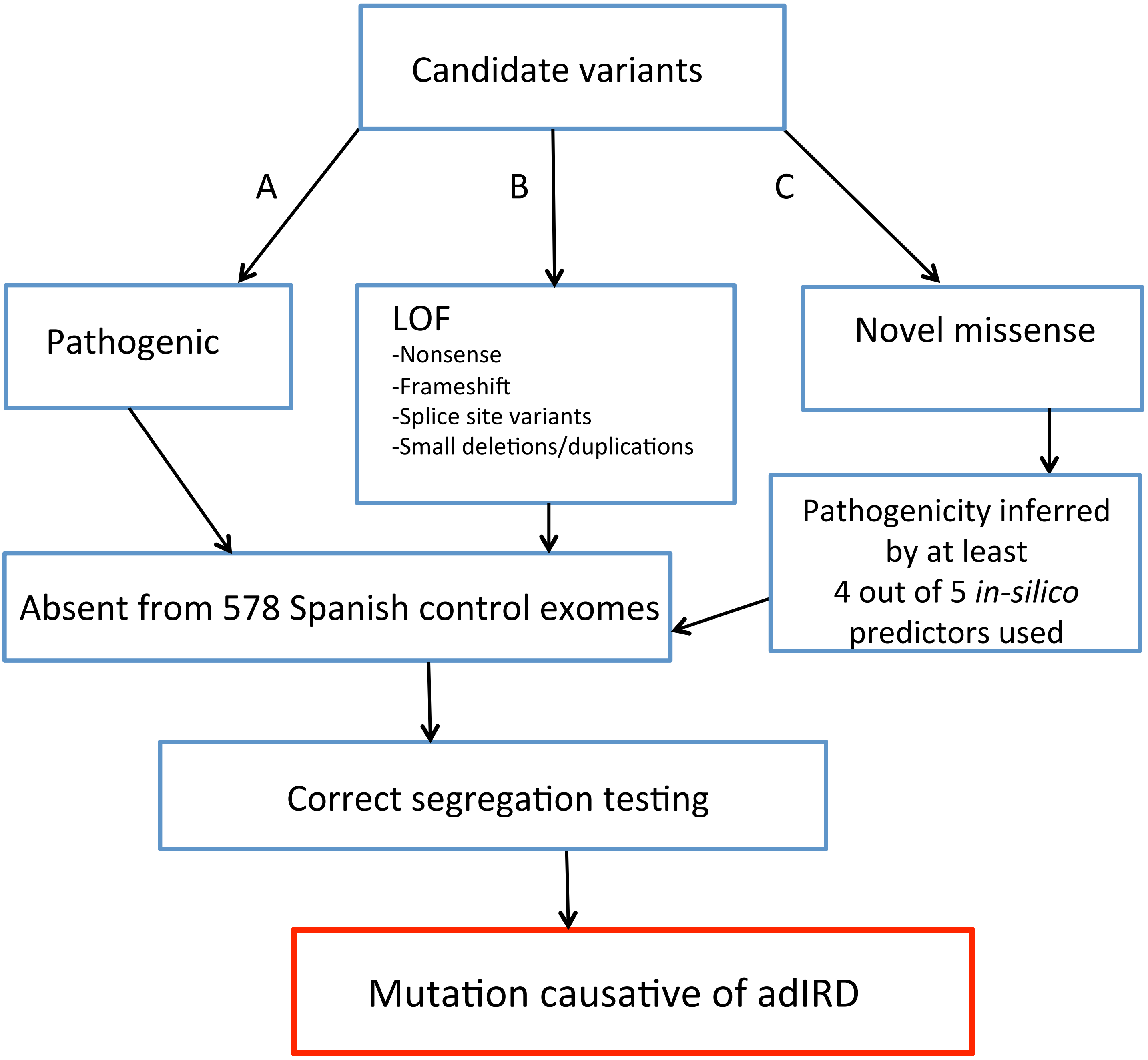

Supplementary Figure S4. Schematic representation of the criteria used to select mutations responsible for autosomal dominant inherited retinal dystrophies. adIRD: Autosomal dominant inherited retinal dystrophies. LOF: loss of function.

Supplementary Table S1

A		DISCARDED VARIANTS				SELECTED VARIANTS						
PATIENT	NUMBER OF VARIANTS PER PATIENT	INTRONIC	SYNONIM	UTR	LOW PATHOGENICITY PREDICTION	PATHOGENIC	HIGH PATHOGENICITY PREDICTION	SPLICING VARIANTS	FRAMESHIFT VARIANTS	STOP CODON LOSS	DELETION	STOP CODON GAIN
5	40	21	12	1	5							1
9	49	24	13	4	7			1				
71	42	20	12	2	6		1		1			
98	51	24	17	3	7							
2	48	18	15	7	8							
16	44	16	14	2	10				2			
19S	48	23	14	4	6		1					
20	50	18	18	3	10				1			
22	38	15	14	3	5	1						
37	45	20	13	4	6	1			1			
39	48	24	15	3	6							
42	47	20	16	2	7		1		1			
43	45	19	15	1	8				2			
48	48	22	14	3	8				1			
64	44	21	11	1	8	1			2			
66	47	20	12	3	7		1		4			
69	50	22	15	4	7		1		1			
70	41	15	13	3	8	1			1			
79	43	16	12	5	7				3			
80	46	18	16	3	7				2			
85	44	19	15	3	6				1			
90	42	15	14	3	9						1	
99	44	20	15	2	5				2			
101	47	23	13	4	4	1			2			
102	45	20	11	4	5	1			4			
105	45	20	16	3	5					1		
113	44	19	13	3	7				2			
133	47	17	16	4	8			1	1			
134	46	23	11	5	6	1						
135	41	15	14	2	9		1					
146	49	23	14	4	6			1	1			
148	43	17	19	2	5							
157	49	19	18	4	6	1	1					

Supplementary Table S1 (continued)

B

MUTATION	PATIENT	GENE	VARIANT TYPE	SANGER SEQUENCED	CONFIRMED	FAMILY SEGREGATION	CONCLUSION
c.1625C>G	5	RP1	stop codon gain	yes	yes		confirmed control
c.937-1G>T	9	RHO	splicing variant	yes	yes		confirmed control
c.259C>G	71	RHO	high pathogenicity prediction	yes	yes		confirmed control
c.415delC	71	KLHL7	frameshift variant	no			false positive in other patient
c.324delA	16	SEMA4A	frameshift variant	yes	no		false positive
c.650delG	16	AIPL1	frameshift variant	yes	no		false positive
c.797C>T	19S	PRPH2	high pathogenicity prediction	yes	yes	correct	causative variant
c.415delC	20	KLHL7	frameshift variant	yes	no		false positive
c.3260C>T	22	SNRNP200	pathogenic	yes	yes	correct	causative variant
c.3260C>T	37	SNRNP200	pathogenic	yes	yes	correct	causative variant
c.415delC	37	KLHL7	frameshift variant	no			false positive*
c.415delC	42	KLHL7	frameshift variant	no			false positive*
c.4555T>C	42	RIMS1	high pathogenicity prediction	yes	yes	incorrect	not causative variant
c.1596delT	43	RPE65	frameshift variant	no			false positive*
c.1670delT	43	PROM1	frameshift variant	yes	no		false positive
c.650delG	48	AIPL1	frameshift variant	no			false positive*
c.1596delT	64	RPE65	frameshift variant	yes	no		false positive
c.1670delT	64	PROM1	frameshift variant	no			false positive*
c.3260C>T	64	SNRNP200	pathogenic	yes	yes	incomplete penetrance	causative variant
c.1596delT	66	RPE65	frameshift variant	no			false positive*
c.1670delT	66	PROM1	frameshift variant	no			false positive*
c.2088delT	66	SNRNP200	frameshift variant	yes	no		false positive
c.415delC	66	KLHL7	frameshift variant	no			false positive*
c.2044C>T	66	SEMA4A	high pathogenicity prediction	yes	yes	incorrect	not causative variant
c.2835A>C	69	PRPF8	high pathogenicity prediction	yes	yes	incorrect	not causative variant
c.324delA	69	SEMA4A	frameshift variant	no			false positive*
c.324delA	70	SEMA4A	frameshift variant	no			false positive*
c.149C>T	70	GUCA1A	pathogenic	yes	yes	incorrect	not causative variant
c.2088delT	79	SNRNP200	frameshift variant	no			false positive*
c.324delA	79	SEMA4A	frameshift variant	no			false positive*
c.650delG	79	AIPL1	frameshift variant	no			false positive*
c.2088delT	80	SNRNP200	frameshift variant	no			false positive*
c.324delA	80	SEMA4A	frameshift variant	no			false positive*
c.324delA	85	SEMA4A	frameshift variant	no			false positive*
c.6974_6994del	90	PRPF8	deletion	yes	yes	correct	causative
c.2088delT	99	SNRNP200	frameshift variant	no			false positive*
c.650delG	99	AIPL1	frameshift variant	no			false positive*
c.1336delA	101	PRPF3	frameshift variant	yes	no		false positive
c.1596delT	101	RPE65	frameshift variant	no			false positive*

c.3260C>T	101	SNRNP200	pathogenic	yes	yes	correct	causative variant
c.1336delA	102	PRPF3	frameshift variant	no			false positive*
c.1596delT	102	RPE65	frameshift variant	no			false positive*
c.1670delT	102	PROM1	frameshift variant	no			false positive*
c.193delA	102	CA4	frameshift variant	yes	no		false positive
c.3260C>T	102	SNRNP200	Pathogenic	yes	yes	incomplete penetrance	causative
c.1045T>C	105	RHO	codon stop loss	yes	yes	n/a	causative
c.2088delT	113	SNRNP200	frameshift variant	no			false positive*
c.6945delG	113	PRPF8	frameshift variant	yes	yes	correct	causative variant
c.937-1G>T	133	RHO	splicing variant	yes	yes	correct	causative variant
c.324delA	133	SEMA4A	frameshift variant	no			false positive*
c.3260C>T	134	SNRNP200	pathogenic	yes	yes	correct	causative
c.568G>A	135	RHO	high pathogenicity prediction	yes	yes	n/a	causative variant
c.666insG	146	RIMS1	frameshift variant	yes	no		false positive
c.937-1G>T	146	RHO	splicing variant	yes	yes	correct	causative variant
c.1961G>T	157	SNRNP200	high pathogenicity prediction	yes	yes	n/a	not causative variant
c.3260C>T	157	SNRNP200	pathogenic	yes	yes	n/a	causative variant

Supplementary Table S1. Variant identification process in each patient analysed. Classification of all variants detected in each patient (**A**). Selection of variants likely involved in adIRD as determined by previous studies or by *in silico* preditors. Only those variants confirmed by Sanger were submitted to segregation analysis (**B**) (for selection criteria used see Supplementary Fig. S4). Asterisks indicate highly repetitive variants, found in several patients and therefore regarded as false positives.

Supplementary Table S2

PATIENT	AGE OF ONSET	SYMTONS AT DIAGNOSIS	VISUAL ACUITY IN 2015 (LogMAR VA RE)	VISUAL ACUITY IN 2015 (LogMAR VA LE)	ESPHERICAL EQUIVALENT RE	ESPHERICAL EQUIVALENT LE	CATARACT (YES, NO, PF -PSEUDOPHAKIA-, APHAKIA)RE	CATARACT (YES, NO, PF -PSEUDOPHAKIC-, AFAQUIC)LE	IOP RE	IOP LE	PALE OPTIC DISC	ARTERIOLAR ATTENUATION	BONE SPICULE PIGMENT	SECTORIAL RP(YES/NO)	EPIRETINAL MEMBRANE (ERM)	MACULAR EDEMA	MACULAR THICKNESS RE(STRATUS OCT)	MACULAR THICKNESS LE(STRATUS OCT)	MACULAR THICKNESS RE(CIRRUS OCT)	MACULAR THICKNESS LE(CIRRUS OCT)	VISUAL FIELD DEGREES	ERG
2	6	NYCTALOPIA	0.2	0.3	-0.25	-0.5	YES	YES	16	16	YES	YES	YES	NO	NO	YES	121	119	169	165	11	ND
16	43	DECREASE VA	0.2	0.1	0	0.125	PP	PP	19	16	YES	YES	YES	NO	YES	YES	146	230			4	NA
19S	13	ASYMPTOMATIC	0	0	0.5	0.875	NO	NO	13	14	NO	NO	YES	NO	NO	NO			266	249	18	NA
20	18	DECREASE VA	1	0.5	-5.25	-3.75	YES	YES	15	15	YES	YES	YES	NO	NO	NO					4	ND
22	45	NYCTALOPIA	0.5	0.8	1.625	2.25	YES	YES	15	17	YES	YES	YES	NO	NO	YES	114	140				ND
37	48	DECREASE VA	4	0.3	-5.375	-4.875	NO	YES	14	14	YES	YES	YES	NO	NO	NO	148	123			0	ND
39	27	DECREASE VA	0	0.2			PP	PP	16	16	YES	YES	YES	NO	NO	YES	244	324			8	ND
42	58	DECREASE VA	2	1.3			PP	YES	16	16	YES	YES	YES	NO	NO	NO	218	165			9	ND
43	49	VISUAL FIELD LOSS	0.2	0.5	-0.25	1.25	NO	YES	17	18	YES	YES	YES	NO	YES	YES	258	320				ND
48	58	NYCTALOPIA	0	0	0.625	-0.875	NO	NO	14	14	YES	YES	YES	NO	NO	NO	225	258	322	336	15	ND
64	22	VISUAL FIELD LOSS	0.1	0.3	-1.5	-1.75	YES	YES	12	12	YES	YES	YES	NO	YES	YES	328	345			10	ND
66	18	VISUAL FIELD LOSS	0.05	0.05	-11.125	-10.125	YES	YES	12	14	YES	YES	YES	NO	NO	NO	171	209			10	ND
69	29	NYCTALOPIA	0.18	0.2	-0.125	-5.75	PP	YES	14	14	YES	YES	YES	YES	NO	YES	191	234			5	ND
89	23	NYCTALOPIA	0.5	0.18	0.125	-1	YES	YES	10	10	YES	YES	YES	NO	NO	NO	121	212			11	ND
90	20	NYCTALOPIA	1.3	1.3	13.25	13.75	AP	AP	8	8	YES	YES	YES	NO	YES	NO	119	253				ND
105	12	NYCTALOPIA	3	2	-1	0.5	PP	PP	16	16	YES	YES	YES	NO								ND
101	37	NYCTALOPIA	0.3	0.3	-2.5	-2	YES	YES	14	14	YES	YES	YES	NO	NO	NO	226	296	312	330	15	ND
102	15	NYCTALOPIA	0.05	0	-5	-4.75	NO	NO	13	15	YES	YES	YES	NO	NO	NO			286	288		ND
113	14	NYCTALOPIA	0.18	0.18			YES	YES	14	14	YES	YES	YES	NO	YES	NO			260	269	5	ND
85	28	DECREASE VA	1	0.4	-8	-2.125	PP	NO	14	14	YES	YES	YES	NO	NO	NO			279	279		NA
80	29	DECREASE VA	0.2	0.5			YES	PP	18	18	YES	YES	YES	NO	SI	YES						NA
133	49	VISUAL FIELD LOSS	0.1	0.05	-0.5	-2.125	PP	YES	16	16	YES	YES	YES	NO	NO	NO			269	252	12	ND
134	44	DECREASE VA	0.2	0.4	-0.25	-0.25	YES	YES	18	18	YES	YES	YES	NO	YES	YES			287	272	10	ND
135	41	NYCTALOPIA	0	0	0.75	-0.625	NO	NO	14	14	NO	NO	YES	YES	NO	NO			267	276	SS	ND
146	37	NYCTALOPIA	0.05	0	0	0.25	NO	NO	17	15	NO	NO	YES	YES	NO	NO			281	294	12	ND
157	62	VISUAL FIELD LOSS	0	0	1.5	-0.625	NO	YES	15	18	YES	YES	YES	NO							5	ND
148	20	NYCTALOPIA	0,8	0,8	-0.25	-0.5	PP	PP	16	15	YES	YES	YES	NO								NA
70	49	PHOTOPHOBIA	0.00	0	0	0.125	NO	NO	14	14	NO	NO	NO	NO	NO	NO					20	NA
79	24	NYCTALOPIA	0.3	0.3	-1	0.5	YES	YES	13	15	YES	YES	YES	NO	NO	NO						

Supplementary Table S2. Clinical features of the patients analysed. Abbreviations; AP: aphakia; ERG: electroretinogram; IOP: intraocular pressure; LE: left eye; N: normal; NA: not available; ND: not detectable; PP: pseudophakia; RE: right eye; SS: superior scotoma.

Supplementary Table S3

AIPL1, BEST1, CA4, CRX, FSCN2, GUCA1A, GUCA1B, GUCY2D, IMPDH1, KLHL7, NR2E3, NRL, OTX2, PITPNM3, PROM1, PRPF3, PRPF31, PRPF6, PRPF8, PRPH2, RDH12, RHO, RIMS1, ROM1, RP1, RP9, RPE65, SEMA4A, SNRNP200, TOPORS, UNC119.

Supplementary Table S3. List of genes analysed.
